# Breast Cancer Detection in Mammography Images Using Deep Convolutional Neural Networks and Fuzzy Ensemble Modeling Techniques

**DOI:** 10.3390/diagnostics12081812

**Published:** 2022-07-28

**Authors:** Ayman Altameem, Chandrakanta Mahanty, Ramesh Chandra Poonia, Abdul Khader Jilani Saudagar, Raghvendra Kumar

**Affiliations:** 1 Department of Computer Science and Engineering, College of Applied Studies and Community Services, King Saud University, Riyadh 11533, Saudi Arabia; aaltameem@ksu.edu.sa; 2 Department of Computer Science and Engineering, GIET University, Odisha 765022, India; chandra.mahanty@giet.edu (C.M.); raghvendra@giet.edu (R.K.); 3 Department of Computer Science, CHRIST (Deemed to be University), Bangalore 560029, India; rameshcpoonia@gmail.com; 4 Information Systems Department, Imam Mohammad Ibn Saud Islamic University (IMSIU), Riyadh 11432, Saudi Arabia

**Keywords:** breast cancer, deep CNN, fuzzy, ensemble, VGG-11, ResNet-164, DenseNet121, Inception V4, Gompertzfunction

## Abstract

Breast cancer has evolved as the most lethal illness impacting women all over the globe. Breast cancer may be detected early, which reduces mortality and increases the chances of a full recovery. Researchers all around the world are working on breast cancer screening tools based on medical imaging. Deep learning approaches have piqued the attention of many in the medical imaging field due to their rapid growth. In this research, mammography pictures were utilized to detect breast cancer. We have used four mammography imaging datasets with a similar number of 1145 normal, benign, and malignant pictures using various deep CNN (Inception V4, ResNet-164, VGG-11, and DenseNet121) models as base classifiers. The proposed technique employs an ensemble approach in which the Gompertz function is used to build fuzzy rankings of the base classification techniques, and the decision scores of the base models are adaptively combined to construct final predictions. The proposed fuzzy ensemble techniques outperform each individual transfer learning methodology as well as multiple advanced ensemble strategies (Weighted Average, Sugeno Integral) with reference to prediction and accuracy. The suggested Inception V4 ensemble model with fuzzy rank based Gompertz function has a 99.32% accuracy rate. We believe that the suggested approach will be of tremendous value to healthcare practitioners in identifying breast cancer patients early on, perhaps leading to an immediate diagnosis.

## 1. Introduction

In both emerging and developing nations, breast cancer is the deadliest disease among women. Breast cancer will be diagnosed 19.3 million times by 2025, as per the World Health Organization (WHO) [1]. Patients may be able to receive appropriate therapy if breast cancer is detected and classified early. Breast cancer is the most prevalent cancer in women, affecting 2.1 million people yearly and is responsible for the majority of cancer-related fatalities in women. In the year 2018, an estimated 627,000 women died from breast cancer [1]. According to a current study released by the National Cancer Registry Program (NCRP), cancer cases in India are predicted to grow by about 20% by 2025, from 13.9 lakhs in 2020 to 15.7 lakhs in 2025 [2]. In high-income countries, age-standardized breast cancer mortality reduced by 40% between 1980 and 2020. Breast cancer mortality has decreased by 2–4 percent every year in countries that have succeeded in decreasing it. If worldwide mortality rates reduced by 2.5 percent every year between 2020 and 2040, 2.5 million breast cancer deaths could be avoided [3]. Breast cancer is a category of diseases in which cells in the breast tissue alter and divide unregulated, leading to a tumor. Most breast cancers develop in the lobules that link the lobules to the nipple. Breast discomfort, changes in breast skin color, creation of a breast mass and changes in breast shape and size are all symptoms of breast cancer. X-rays, magnetic imaging and ultrasound are all commonly used to discover breast cancer [4]. Mammography, which employs low-dose X-rays to produce pictures, is one of the most effective treatments for detecting breast cancer early [5]. Researchers from all around the world are working on deep learning models for breast cancer screening based on medical imaging. Breast cancer screening sometimes requires a good visual examination to identify any irregularities, such as lumps, that may signify disease. After these nodules have been found, relevant measurements may be obtained to help clinicians in determining the presence or absence of malignant tissue. Mammography may detect more subtle signs, including structural distortion and bi-lateral asymmetry, as well as more obvious abnormalities such as calcification and masses. A nodule, mass or densities are all possible abnormalities in mammography. Nevertheless, not all anomalies are malignant. For example, a smooth bounded bulge is often benign. A starburst-shaped, irregularly bordered tumor, on the other hand, might be malignant, and a biopsy is necessary to confirm this [6]. Breast cancer cells may move to lymph glands and cause injury to lungs and other regions of body. The most prevalent cause of breast cancer is a malfunction of the milk-producing ducts, often known as invasive ducts. It may also begin in the breast’s lobules, a kind of glandular tissue, or other cells and tissues. Environmental, hormonal and lifestyle factors have all been linked to an increased risk of breast cancer, according to researchers. Due to the unequal function and massive multiplication of abnormal cells, it creates a tumor in the breast and results in death [7]. These mammography images are examined by radiologists in order to diagnose breast cancer. Nevertheless, radiologists’ assessments on the existence of breast cancer may vary owing to variations in their past experiences and understanding. As a result, a deep-CNN-based breast cancer detection approach may be employed to boost radiologist confidence and serve as a second opinion in the diagnosis of breast cancer. The present study includes many studies on several deep CNN models for detecting breast cancer using mammography images.

Naji et al. [8] employed Decision Tree (DT), Nave Bayes (NB), Simple Logistic, and advanced ensembles technologies such as Majority Voting and Random Forest (RF) approach to diagnose breast cancer with 98.1% accuracy and a 0.01 percent error rate. Chakravarthy et al. [9] proposed an improved crow search optimized extreme learning machine (ICSELM) technique and achieved an accuracy of 98.26%, 97.193%, and 98.137% for the INbreast, DDSM, and MI-AS datasets, respectively. Faisal et al. [10] used and compared individual classifiers such as the Neural Network, MLP, NB, SVM, Gradient boosted Tree (GBT) and DT. The use of MV-based ensembles and RF is also looked at. The author obtained 90% accuracy with the GBT ensemble. A back propagation neural network (BPNN) classification model was used by Mughal et al. [11]. In the early-stage DDSM and MIAS datasets, their system properly recognized the tumor with 99% accuracy. Wei et al. [12] proposed a BiCNN model, which was proven to be 97.97% accurate. Khuriwal et al. [13] used logistic regression and Artificial Neural Network (ANN) fused with a voting algorithm technique for diagnosing breast cancer and achieved 98% accuracy. Thuy et al. [14] used a hybrid deep learning model that incorporated VGG19 and VGG16 models, as well as a generative adversarial network (GAN) to improve classification performance and reached 98.1% accuracy. Bhowal et al. [15] used the Coalition Game and Information Theory to present Choquet Integral-based deep CNN models for a four-class problem in breast cancer histology and achieved 95% accuracy. Khan et al. [16] recommended a novel CNN model combined with various transfer learning algorithms and achieved 97.67% accuracy. Muduli et al. [17] promote a novel deep CNN model that yields 96.55% accuracy. Furthermore, several studies [18,19,20,21,22,23,24,25] used several deep CNN models to diagnose breast cancer.

## 2. Motivation and Contributions

According to a study of the relevant literature, few researchers worked on fuzzy ensemble techniques linked with deep CNN models. An ensemble method is a machine learning strategy that blends numerous base models into a single best prediction model. The results of various models are merged to boost overall performance. Numerous deep CNN approaches are integrated into a single predictive model to boost overall performance and predictions while minimizing bias and variation. In addition, merging deep transfer learning models with fuzzy ensemble techniques may boost the accuracy and robustness of a detection system. In this work, we employed the Gompertz function to construct a fuzzy ranking algorithm. The benefit of such fusion is that it provides the final prediction for each sample using adaptive weights relay on each classifier confidence scores used to create the ensemble. The Gompertz function was developed on the notion that as an individual aged, mortality reduces exponentially until it approaches an asymptote. It might be useful for fusing the confidence scores of classifiers in a complicated image classification issue, in which the confidence score for a prediction category by a classifier ever achieves absolute zero value but rather some lesser value.

The study looked at the following objectives:

1. The aim of our study is to build a fuzzy ensemble methodology that takes breast mammography images as input. Initially, we employed multiple pre-trained deep CNN models to diagnose cancer in mammography images, including VGG-11, ResNet-164, DenseNet121, and Inception V4.
2. We used dense and softmax layers to extract characteristics and categorize mammography pictures utilizing pre-trained deep CNN models. An ensemble technique was utilized to combine the decision scores of the aforementioned models.
3. Using a re-parameterized Gompertz function, the ensemble approach delivers fuzzy rankings to the component classifiers. Fuzzy fusion outperforms traditional ensemble algorithms because it uses adaptive priority depending on the classifiers’ confidence levels for each sample to be predicted.
4. The Gompertz function displays exponential growth before saturating to an asymptote, and that is beneficial for assembling the decision values of deep CNN methodologies, since the decision value of a class forecasted by a classifier typically reaches zero.
5. The framework’s efficiency was assessed using recall, precision, F1-Score, specificity, and sensitivity. The gathered results beat the present methodologies by a significant amount.

We used the accuracy of each classifier to estimate the fuzzy membership values of each classifier when using the Gompertz function with other advanced models such as the Sugeno Integral and the Weighted Average. This kind of fusion seems to have the benefit of employing adaptive weights depending on the sample’s confidence values to generate each sample’s final prediction.

The following is how the rest of the article is structured. Section 2 discusses the motive and contribution. The materials and methods are described in Section 3. The experimental findings, evaluations, and comparative analyses are presented in Section 4. Section 5 contains the discussion and conclusion.

## 3. Materials and Methods

### 3.1. Deep CNN Models

#### 3.1.1. VGG-11

**Figure 1:**
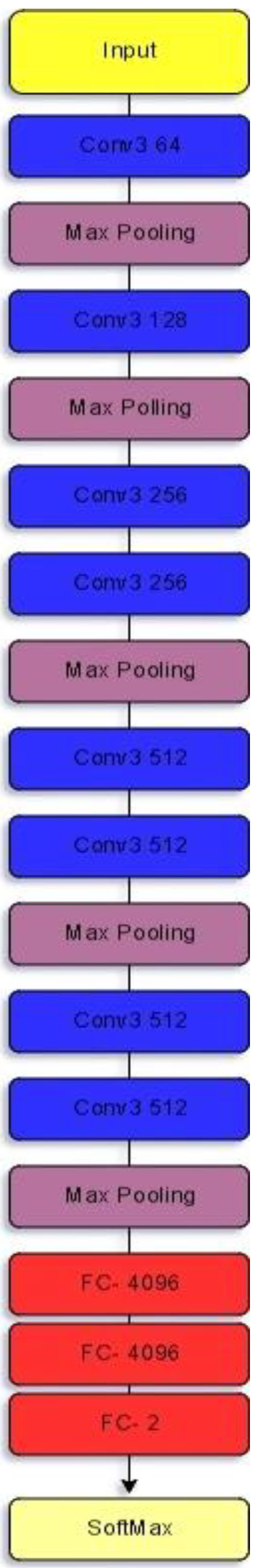
VGG-11 architecture.

The Visual Geometry Group (VGG) [26] models are one of the deep CNN models. The VGG group emphasizes the importance of a CNN model’s depth for visual depictions and correct application to a broad variety of computer vision classification applications. By reducing the size of the convolution filters to 3 × 3 kernels, it was feasible to add many weight layers ranging from 16 to 19 layers. VGG-11 is made up of eleven weight layers, eight convolution layers, and three fully linked layers. The pooling layer’s window size is 2 × 2 and the stride size is 2. It is used to minimize the size of the convoluted feature image while also ensuring the model’s translation invariance. Finally, a softmax classifier layer categorized it. All hidden layers are equipped with the RELU function as activation function. Figure 1 depicts the VGG-11 CNN architecture.

#### 3.1.2. ResNet-164

ResNet-164 combines the basic residual structure with 164 deep layers. It uses several convergence filters that have been trained on millions of pictures to avoid degradation [27]. ResNet-164 is the outcome of adding the residual block to the model, which feeds residual data to the subsequent layers. This is no longer a ResNet-164 classic model feature. It was developed by a Microsoft research team to prevent gradient convergence from reaching zero in very deep networks. The ResNet-164’s operation is simple: a few layers collect and activate the activation function in front of the input of the current activation function. As a result, an output is created even if the result of the linear transform of the layer on which the operation is performed is 0.

**Figure 2:**
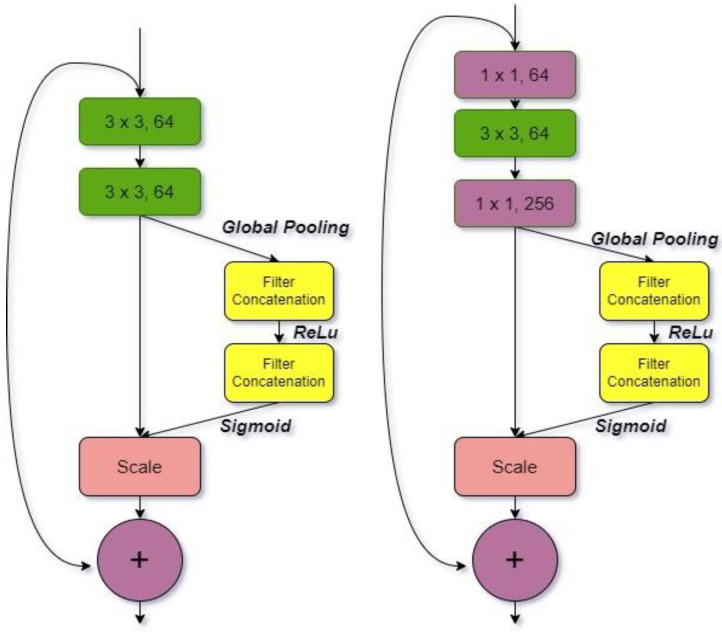
ResNet-164 architecture.

The ResNet-164 network was built by combining shortcuts with the standard network seen in Figure 2, which is formed up of leftover blocks. The value is received as input and sent via the residual block’s convolution. A sequence of activation convolutions is generated, as well as a function 𝑓 (𝑥). $h\left(x\right)=f\;\left(x\right)+x$ is then formed by adding the original input value of x to the function $f\;\left(x\right)$. The function $h\;\left(x\right)$ is equivalent to the function $f\;\left(x\right)$ in the standard convolution operation [28]. The original data are also incorporated once the convolution method is applied to this network’s input. Figure 2 depicts the ResNet-164 architecture.

#### 3.1.3. DenseNet121

**Figure 3:**
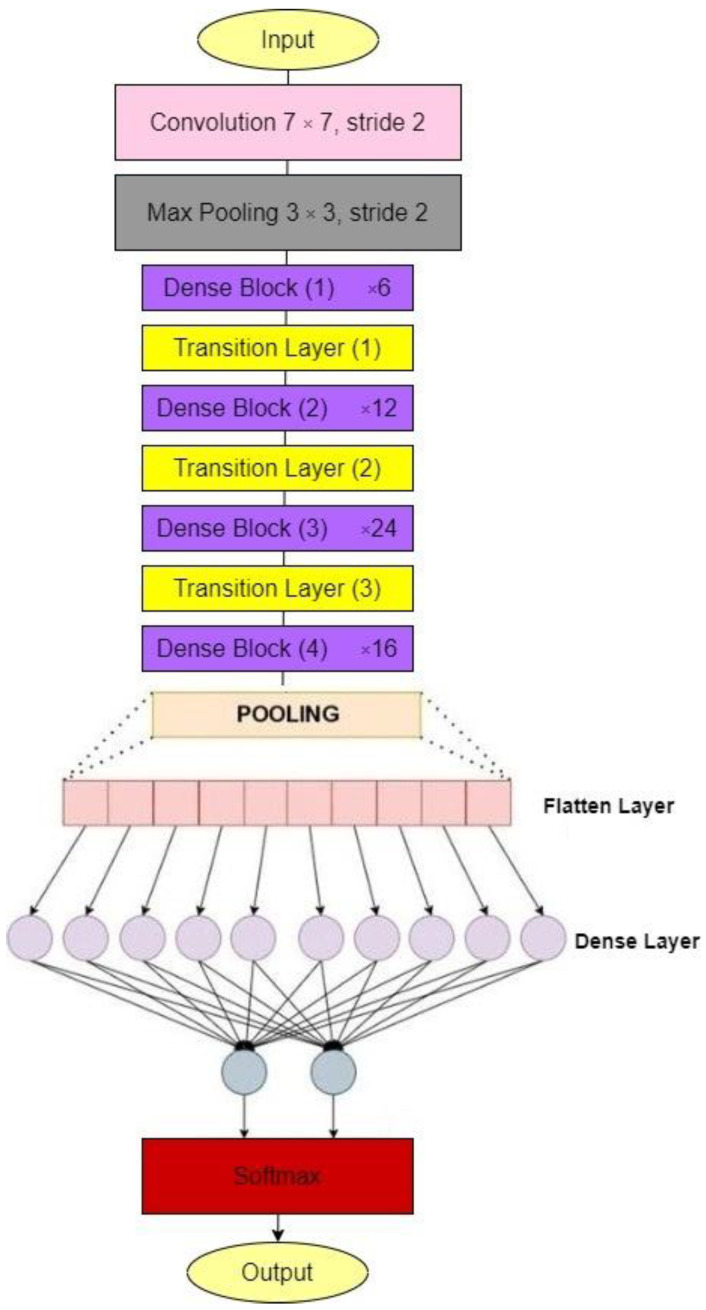
DenseNet121 architecture.

Each layer in the Dense Net [29] design is linked to every other layer. It is utilized to solve the issue of gradient vanishing. $L\left(L+1\right)/2$ direct connections exist in this model with L layer. It connects the output and input feature maps, giving each layer access to all preceding layers’ collective knowledge. This study helps to solve the vanishing gradient problem, reduce the number of parameters, and introduce the idea of feature reuse. Because of its dense connection architecture, it requires less parameters than typical convolutional networks because it does not require relearning excessive feature mappings. The network is organized into dense blocks, with the feature map dimensions remaining constant within each block but the number of filters varying between them. It provides various significantly lowered number of parameters, the reuse of features, and the mitigation of the vanishing gradient. Figure 3 depicts the DenseNet121architecture.

#### 3.1.4. Inception V4

**Figure 4:**
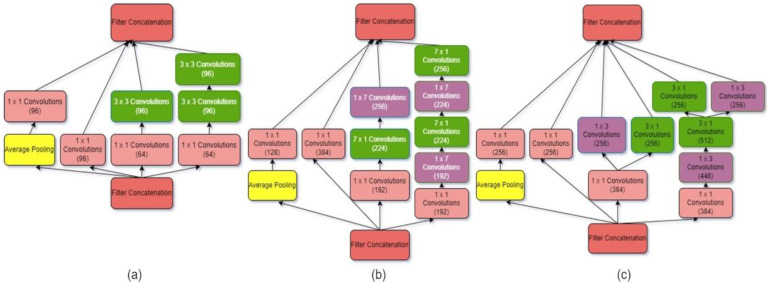
Details of Inception V4 architecture with (**a**–**c**) layers.

**Figure 5:**
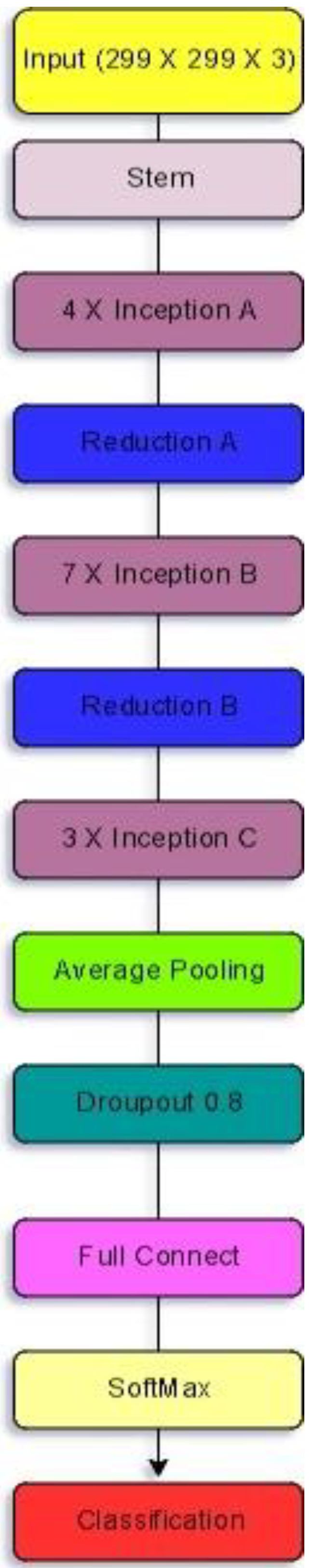
Inception V4 architecture.

Inception V4 is a deep CNN architecture that improves on earlier inception family generations by simplifying the architecture, adding a stem layer, and utilizing more inception modules than Inception v3 [30]. Unlike prior versions of Inceptions, which required various replicas to fit in memory, this model may be trained without partitioning replicas. Memory optimization on back propagation is used in this design to decrease memory requirements. Internal layers can determine which filter size is most useful to acquiring the essential information thanks to Inception layers. Between the three Inception modules, the Reduction modules serve as pooling layers. Four Inception A layers, seven inception B layers, and three inception C layers are depicted in Figure 4. The overall system configuration presented in Figure 5.

### 3.2. Data Set

For testing purposes, we employed multi-modal breast cancer datasets such as mammography images. Four widely used and publicly available mammography databases are included in this study: the Breast Cancer Digital Repository (BCDR) [31], the Mini Mammographic Image Analysis Society (Mini-MIAS) [32], INbreast [33], and the Digital Database for Mammography Screening (DDSM) [34]. We used an equal number of normal, benign, and malignant mammography images from the whole dataset. Each class has 1145 images. Using normal, benign, and malignant mammography pictures, the deep learning models Tensor Flow and Keras were trained to identify whether or not a person had breast cancer. The data were separated into two groups: 30% for the test set and 70% for the training set, with the same groups used for all models.

### 3.3. Experimental Environment

Our experiment is built in Python and runs on Google Colaboratory, a machine with a GPU and 12 GB of RAM that runs the Keras deep learning framework backend. In mammography images of breast cancer, our approach has been deployed to three-class classification concerns (normal, benign, and malignant). We use the same set of hyperparameters to train all four deep CNN models on the mammography images dataset. We resize the images to 224 × 224 × 3 throughout the input process. It is important to adjust the images to a size that is compatible. As a result, black borders are applied to the edges of the images to verify that they correspond to the square input. The original model’s architecture has been kept, excluding the layers that follow the convolutional layers.

After the feature extractors, the weights of the convolutional layers are frozen, and more layers, such as the max pooling layer, fully connected layers, dense layers, etc., are added according to the various deep CNN models. The softmax activation function is included in the last layer of each CNN model. The output of this layer represents a probability distribution over the predicted output classes, which we refer to as the confidence score generated by the classifier. To avoid overfitting of the deep CNN models, we utilize 100 epochs and a learning rate of 1 × 10^−4^. We utilized ADAM as our optimizer for compilation, and after extracting features from the pre-trained models, we employed two dense layers with 4096 neurons each as part of the classifier with ReLU as the activation function. The last layer consists of three Softmax output nodes.

### 3.4. Proposed Framework

**Figure 6:**
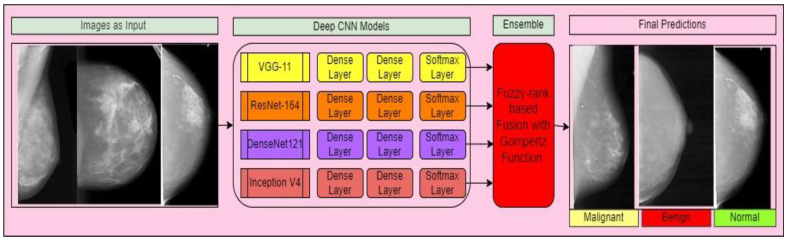
Workflow of the proposed framework.

**Figure 7:**
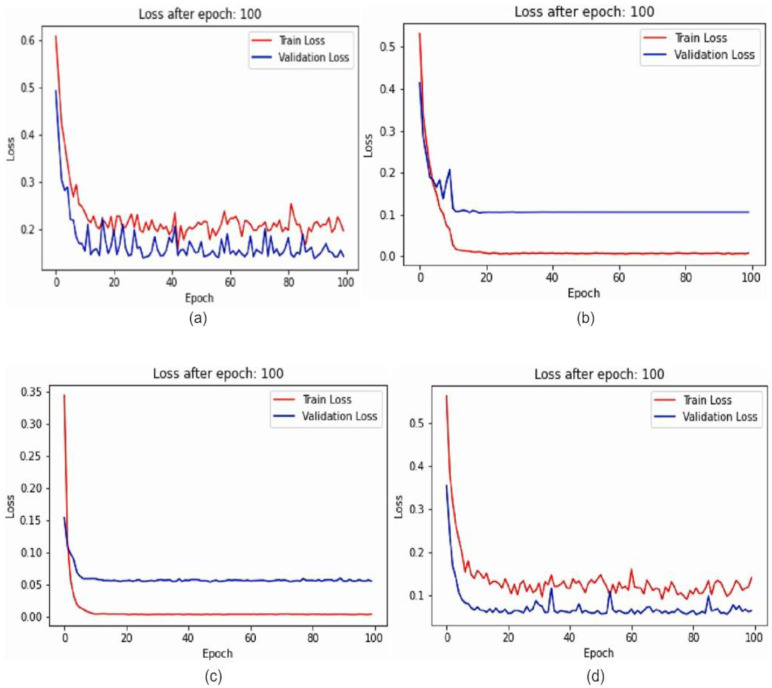
Loss graph for (**a**) ResNet-164, (**b**) VGG-11, (**c**) DenseNet121 and (**d**) Inception V4.

The suggested framework for breast cancer classification from mammography images is divided into two stages: producing confidence values from various models and fusing the decision scores utilizing fusion of fuzzy rank and Gompertz function to create final predictions. Figure 6 depicts the workflow of the proposed system. Figure 7 shows the training loss and validation graphs for four deep CNN models.

### 3.5. Ensemble Technologies

Ensemble models incorporate the best features of all participating classifiers, enabling them to outperform single models. Numerous advanced ensemble techniques have arisen throughout time, and some of them have been examined in this study to demonstrate the recommended ensemble’s superiority over existing methods.

#### 3.5.1. Weighted Average (WA)

An approach for computing the fuzzy weighted average was presented by Dong and Wong [35]. The weighted average approach averages the final prediction output from numerous weak learning devices. Instead of using serial and parallel structures, the weighted average technique assigns various weights to each learner to arrive at the final findings. Let $W_{1},\;W_{2}\ldots \ldots \;W_{n}$ and $A_{1},\;A_{2}\;\ldots \ldots \;\;A_{n}$ be the fuzzy numbers defined on the universes $Z_{1},\;Z_{2}\;\ldots \ldots \;Z_{n}$ and $X_{1},\;X_{2}\;\ldots \ldots \;\;X_{n}$, respectively. If f is a function which maps from $\;Z_{1}\times Z_{2}\times \ldots \times Z_{n}\times X_{1}\times X_{2}\times \ldots \times X_{n}$ to the universe Y, then the fuzzy weighted average y is represented as

$$y=f(x_{1\;},\;x_{2\ldots \ldots \;\;},\;x_{n},\;w_{1\;},\;w_{2\ldots \ldots \;\;},\;w_{n})=(x_{1\;}w_{1\;}+x_{2\;}w_{2\;}+\ldots \ldots +x_{n}w_{n})\;/(w_{1\;}+w_{2\;}+\ldots \ldots +w_{n}),$$

where, for each $i=1,\;2\;\ldots \ldots n,\;x_{i}\in X_{i}w_{i}\in Z_{i}$ and $\left(w_{1\;}+w_{2\;}+\ldots \ldots +w_{n}\right)$ > 0.

#### 3.5.2. Sugeno Integral (SI)

Takagi-Sugeno [36] is a fuzzy inference approach for generating fuzzy rules from a particular input–output dataset. The inputs are hazy, but the result is crystal clear. Takagi Sugeno uses a weighted average to calculate the crisp output. This technique is more computationally efficient and may be used alongside optimization and adaptive methods.

#### 3.5.3. Fuzzy-Rank-Based Fusion with Gompertz Function (FRGF)

The Gompertz function is used to identify time series that expand slowly at the start and conclusion of a period. It was developed to represent the rate of child mortality as they became older, but it is now extensively used in biology. A population’s growth, a malignant tumor’s development, a bacterial colony’s growth, and the number of persons impacted during an epidemic may all be explained using the Gompertz function. In the classic ensemble technique, the classification scores of all component models are given equal weight, whereas the classifiers are given pre-computed weights. The fundamental problem with such an ensemble is the production of static weights, which are hard to change after the test samples have been classified. On the other hand, the suggested fuzzy rank ensemble technique evaluates each base classifier’s predictions scores for each unique test case separately. Improved and more accurate classification scores may be produced using this above methodology. There is no need to alter the weights for various test datasets since this is a dynamic process.

Gompertz function [37] is written as:

(1) $$f\left(t\right)=ae^{-e^{d-kt}}$$

where *d* sets the *x*-axis displacement, *a* is an asymptote, *e* is the Euler’s Number, and *k* is used for y-scaling.

The fundamental reason for utilizing a fuzzy rank method is because, unlike classic ensemble approaches such as the weighted average rule and the average rule, each classifier’s confidence in its predictions is prioritized for each individual test case.

In order to diagnose breast cancer from mammography pictures, the re-parameterized Gompertz algorithm [38] is utilized to build the fuzzy ranks of each deep CNN classifier. We have X number of prediction scores for each picture database’s test split if X is the number of component models. As previously mentioned, we used four transfer learning models; hence, X=4. For each picture, suppose there are X number of decision scores of classifiers $\left\{DC^{1},\;DC^{2},\;\ldots DC^{X}\right\}$ for each image. If Y is the dataset’s number of classes, then:

(2) $$\sum_{y=1}^{Y}DC_{y}^{\left(n\right)}=1$$

where n=1, 2, 3…X.

**Figure 8:**
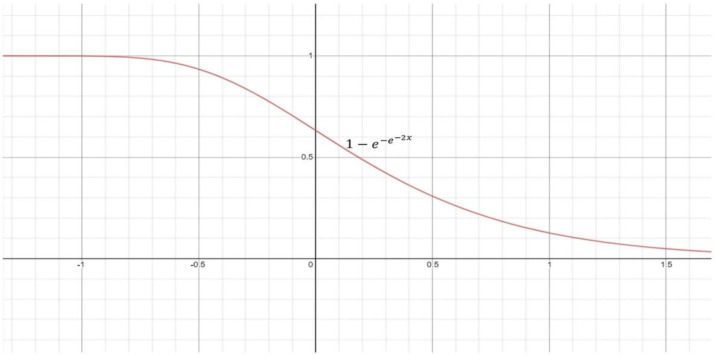
Showing the re-parameterized Gompertz function.

When creating the fuzzy rankings, the decision scores represented by DC in Equation (2) of each class for each supplied data are taken into consideration. $\;DC_{y}^{\left(n\right)}$ is the output of a softmax function. Figure 8 depicts the recommended re-parameterized Gompertz function, in which the independent variable ‘*x*’ signifies a classifier’s projected confidence score for a test sample.

The confidence scores are used to create the fuzzy rankings for all samples in the dataset that correspond to distinct classes. The Gompertz function generates the fuzzy rank for a class *y* using the confidence ratings of the $k^{th}$ classifier, as shown in Equation (3):

(3) $$FR_{y}^{\left(n\right)}=1-e^{-e^{-2*DC_{y}^{\left(n\right)}}}$$

where y=1, 2, 3….Y and n=1, 2, 3….X.

The value of $FR_{y}^{\left(n\right)}$ ranges from 0.127 to 0.632, while the lowest value 0.127 corresponding to higher confidence results in a lower value of rank. Fuzzy rank sum (FRSum) and the complement of confidence factor sum $\left(CCFSum\right)$ are determined as in Equations (4) and (5), respectively. If $M^{\left(i\right)}$ denotes the top, most m ranks, i.e., rankings 1, 2, …, m, belonging to class y.

A penalty value of $P_{y}^{FR}$ and $DC_{y}^{\left(n\right)}$ is placed on the relevant class if the label y does not quite fall inside the top M classes. The $P_{y}^{FR}$ value is 0.632, which is obtained by putting $DC_{y}^{\left(n\right)}$ = 0 in Equation (3), and the $P_{y}^{DC}$ value is set to zero. The penalty values prevent class *y* from being a probable winner. As stated in Equation (6), the final decision scores FDC for the data instance Z are generated by multiplying $\left(FRSum\right)$ and $\left(CCFSum\right)$ and evaluating the lowest value across all of the classes:

(4) $$FRSum_{y}=\overset{X}{\underset{n=1}{\sum }}\left(\begin{array}{c} FR_{y}^{\left(n\right)},\;ifFR_{y}^{\left(n\right)}\in M^{\left(i\right)}\\ P_{y}^{FR},\;Otherwise \end{array}\right)$$

(5) $$CCFSum_{y}=\frac{1}{X}\overset{X}{\underset{n=1}{\sum }}\left(\begin{array}{c} DC_{y}^{\left(n\right)},\;ifFR_{y}^{\left(n\right)}\in M^{\left(i\right)}\\ P_{y}^{DC},\;Otherwise \end{array}\right)$$

(6) $$class\left(Z\right)=min\left\{\left(FRSum_{y}\right)*\left(CCFSum_{y}\right)\right\}$$

where y=1, 2, 3….Y.

## 4. Experimental Results and Evaluations

**Figure 9:**
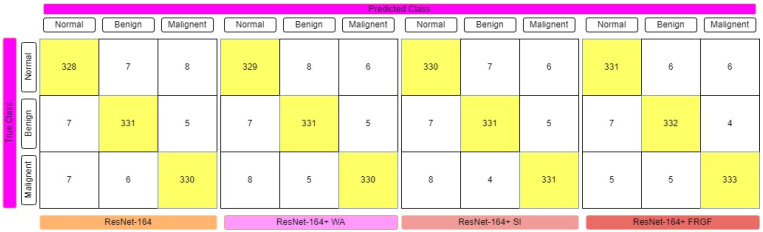
Confusion matrix representation of ResNet-164 deep CNN model with various fuzzy ensemble technologies. ResNet-164, ResNet-164+ WA, ResNet-164+ SI, and ResNet-164+ FRGF showed 96.11%, 96.21%, 96.40%, and 96.79% of accuracy, respectively.

**Figure 10:**
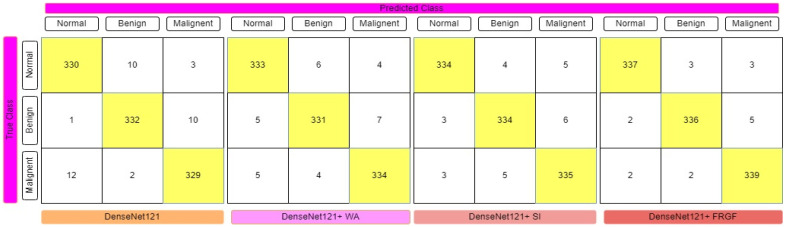
Confusion matrix representation of VGG-11 deep CNN model with various fuzzy ensemble technologies. VGG-11, VGG-11+ WA, VGG-11+ SI, and VGG-11+ FRGF showed 96.21%, 96.70%, 97.08%, and 97.67% accuracy, respectively.

**Figure 11:**
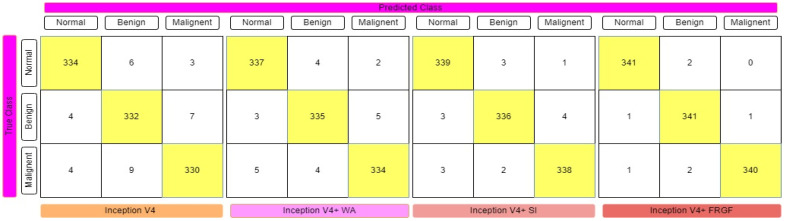
Confusion matrix representation of DenseNet121 deep CNN model with various fuzzy ensemble technologies. DenseNet121, DenseNet121+ WA, DenseNet121+ SI, and DenseNet121+ FRGF showed 96.31%, 96.99%, 97.47%, and 98.35% accuracy, respectively.

**Figure 12:**
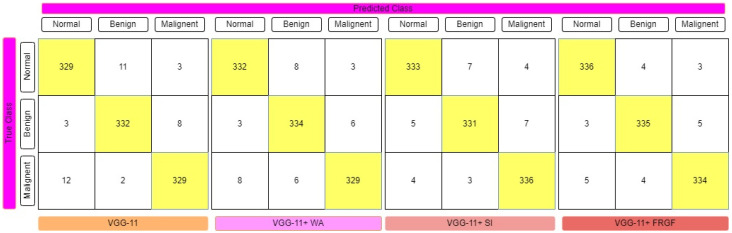
Confusion matrix representation of Inception V4 deep CNN model with various fuzzy ensemble technologies. Inception V4, Inception V4+ WA, Inception V4+ SI, and Inception V4+ FRGF showed 96.79%, 97.76%, 98.45%, and 99.32% accuracy, respectively.

The fuzzy-logic-based ensemble works particularly well when assigning weights to the predictions for rendering a final judgment on the classification of an image, since the confidence in a classifier’s prediction is taken into consideration for each sample when assigning weights to the predictions. Table 1 displays the results of the ensemble built using the four deep CNN models, showing that the Gompertz function fused with fuzzy rank beats the others outstandingly. Sugeno Integral’s fuzzy-integrals-based ensemble approach values come closer to the recommended ensemble strategy. The Weighted Average ensemble is a static approach in which the weights of the classifiers cannot be changed dynamically at prediction time, and it also performs well. The Fuzzy-fusion-based solutions may be able to address this problem by prioritizing confidence scores, resulting in a more effective ensemble method. The sensitivity, specificity, accuracy, and F1-Score of each model were evaluated, with the results displayed in Table 1. The confusion matrices of the VGG-11, ResNet-164, DenseNet121, and Inception V4 employing fuzzy ensemble techniques are shown in Figure 9, Figure 10, Figure 11 and Figure 12, respectively. Table 2 compares the performance of multiple transfer learning approaches with the proposed mammography-image-based methodology.

## 5. Discussion and Conclusions

Breast cancer has become the main cause of mortality among women all over the globe. Breast cancer identification and treatment at an early stage is predicted to decrease the need for surgery and raise the survival rate. Transfer learning on supplementary advanced CNNs was initially applied to produce decision scores from medical pictures. Then, a fuzzy ensemble framework was constructed employing the Weighted Average, Sugeno Integral, and Fuzzy-rank-based Gompertz function to aggregate CNN decision scores using an adaptive combination approach dependent on the confidence of each decision score. The suggested framework may be applied to boost the predicted accuracy of current approaches that, in the overwhelming majority of instances, do not apply a classifier fusion strategy. The fuzzy integral based ensemble technique we used has an influence on the dynamic evaluations of each classifier’s confidence. The findings from the complementary set of classifiers are merged using fuzzy ensemble techniques, which dynamically modify weights to the component deep CNNs depending on the confidence ratings of their predictions. Extensive testing on a range of datasets using a number of measurements reveals the resilience of our method, which frequently surpasses the state-of-the-art in the area. For breast cancer, the suggested framework employed an ensemble model employing the Gompertz function and attained a three-class classification accuracy of 99.32%. It also works well on the overwhelming majority of datasets in the field. We have also shown how to apply fuzzy rank fusion using decision values acquired from various deep CNN methodologies to diagnose breast cancer.

In the future, the proposed approach might be extended to breast tissue localization and segmentation to enable medical professionals in better disease identification. A next goal would be to test our model on a more demanding breast cancer picture dataset that could help us show the durability of the model. We want to apply this strategy to other aspects of healthcare where it may benefit the biomedical community as a whole.

## Figures and Tables

**Table 1:** Performance indicators for a variety of deep CNN models using fuzzy ensemble approaches based on testing datasets.

Models	Class	Precision (%)	Recall (%)	Specificity (%)	F1-Score (%)	Accuracy (%)
ResNet-164	Normal	0.959	0.956	0.980	0.958	96.11
	Benign	0.962	0.965	0.981	0.964	
	Malignant	0.962	0.962	0.981	0.962	
ResNet-164+ Weighted Average	Normal	0.956	0.959	0.978	0.958	96.21
	Benign	0.962	0.965	0.981	0.964	
	Malignant	0.968	0.962	0.984	0.965	
ResNet-164+ Sugeno Integral	Normal	0.957	0.962	0.978	0.959	96.40
	Benign	0.968	0.965	0.984	0.966	
	Malignant	0.968	0.965	0.984	0.966	
ResNet-164+ Fuzzy rank based Gompertz function	Normal	0.965	0.965	0.983	0.965	96.79
	Benign	0.968	0.968	0.984	0.968	
	Malignant	0.971	0.971	0.985	0.971	
VGG-11	Normal	0.956	0.959	0.978	0.958	96.21
	Benign	0.962	0.968	0.981	0.965	
	Malignant	0.968	0.959	0.984	0.963	
VGG-11+ Weighted Average	Normal	0.968	0.968	0.984	0.968	96.70
	Benign	0.960	0.974	0.980	0.967	
	Malignant	0.973	0.959	0.987	0.966	
VGG-11+ Sugeno Integral	Normal	0.974	0.968	0.987	0.971	97.08
	Benign	0.971	0.965	0.985	0.968	
	Malignant	0.968	0.980	0.984	0.974	
VGG-11+ Fuzzy rank based Gompertz function	Normal	0.977	0.980	0.988	0.978	97.67
	Benign	0.977	0.977	0.988	0.977	
	Malignant	0.977	0.974	0.988	0.975	
DenseNet121	Normal	0.962	0.962	0.981	0.962	96.31
	Benign	0.965	0.968	0.983	0.967	
	Malignant	0.962	0.959	0.981	0.961	
DenseNet121+ Weighted Average	Normal	0.971	0.971	0.985	0.971	96.99
	Benign	0.971	0.965	0.985	0.968	
	Malignant	0.968	0.974	0.984	0.971	
DenseNet121+ Sugeno Integral	Normal	0.982	0.974	0.991	0.978	97.47
	Benign	0.974	0.974	0.987	0.974	
	Malignant	0.968	0.977	0.984	0.972	
DenseNet121+ Fuzzy rank based Gompertz function	Normal	0.988	0.983	0.994	0.985	98.35
	Benign	0.985	0.980	0.993	0.982	
	Malignant	0.977	0.988	0.988	0.983	
Inception V4	Normal	0.977	0.974	0.988	0.975	96.79
	Benign	0.957	0.968	0.978	0.962	
	Malignant	0.971	0.962	0.985	0.966	
Inception V4+ Weighted Average	Normal	0.977	0.983	0.988	0.980	97.76
	Benign	0.977	0.977	0.988	0.977	
	Malignant	0.979	0.974	0.990	0.977	
Inception V4+ Sugeno Integral	Normal	0.983	0.988	0.991	0.985	98.45
	Benign	0.985	0.980	0.993	0.982	
	Malignant	0.985	0.985	0.993	0.985	
Inception V4+ Fuzzy rank based Gompertz function	Normal	0.994	0.994	0.997	0.994	99.32
	Benign	0.988	0.994	0.994	0.991	
	Malignant	0.997	0.991	0.999	0.994	

**Table 2:** Comparison of the performance of several deep CNN models with the suggested breast cancer detection methodologies.

Authors	Technology	Accuracy (%)
Naji et al. [8]	DT, NB, Simple Logistic with RF, and majority-voting-based ensembles	98.1
Faisal et al. [10]	GBT with Majority voting and RF-based ensembles	90
Wei et al. [12]	BiCNN model	97.97
Khuriwal et al. [13]	Voting algorithm ensemble with logistic regression and ANN	98
Bhowal et al. [15]	Choquet-Integral-based deep CNN models using Coalition Game and Information Theory	95
Rajaraman et al. [20]	Stacked ensemble	98.07
Lakhani et al. [21]	Weighted Average	99.14
Proposed Model	Inception V4 with Fuzzy-rank-based Gompertz function ensemble	99.32

## Data Availability

The data presented in this study are available on request from the corresponding author.
